# Factors underlying the high occupational risk of healthcare personnel for COVID-19 infection

**DOI:** 10.31744/einstein_journal/2024AO0433

**Published:** 2024-05-20

**Authors:** Priscila Montesano Cunha Crispim, Julia Yaeko Kawagoe, Ana Cristina Rosseti, Fernando Gatti de Menezes

**Affiliations:** 1 Faculdade Israelita de Ciências da Saúde Albert Einstein Hospital Israelita Albert Einstein São Paulo SP Brazil Faculdade Israelita de Ciências da Saúde Albert Einstein, Hospital Israelita Albert Einstein, São Paulo, SP, Brazil.; 2 Hospital Municipal da Brasilândia São Paulo SP Brazil Hospital Municipal da Brasilândia, São Paulo, SP, Brazil.

**Keywords:** Occupational risks, SARS-CoV-2, COVID-19, Coronavirus infections, Health personnel, Transmission

## Abstract

**Objective:**

To verify the rate of COVID-19 infection among healthcare personnel at high and low risk of COVID-19 infection and identify the underlying risk factors.

**Methods:**

This cross-sectional study was conducted between December 1, 2020 and February 28, 2021. Associations were verified between the levels of risk (high or low) of occupational COVID-19 infection and participant characteristics using the World Health Organization risk assessment questionnaire and adjusted using logistic regression models in single and multiple approaches.

**Results:**

Of the 486 participants, 57.4% were classified as having a high occupational risk for SARS-CoV-2 infection, with a diagnosis rate of 12.1%. The factors identified in the multivariate analysis for high occupational risk were age up to 29 years (odds ratio [OR] = 2.7, 95% confidence interval [95%CI] = 1.63-4.47), monthly family income greater than eight times the basic salary (OR= 1.8, 95%CI= 1.07-3.16), and healthcare personnel who did not participate in initial training to work in the area of patients with COVID-19 infection (OR= 2.39, 95%CI= 1.53-3.75).

**Conclusion:**

Encouraging training for occupational infection prevention is very important to reduce the impact of infectious diseases on healthcare personnel, especially young health professionals.

## In Brief

Crispim et al. demonstrated the independent risk factors for acquiring COVID-19 among healthcare personnel. They also showed the importance of infection prevention training to avoid acquiring COVID-19 in this population.

## Highlights

COVID-19 infection among healthcare personnel has impacted the workforce in hospitals.

Knowledge of the risk factors for COVID-19 infection is important for disease prevention measures.

Failure to train healthcare personnel is an important risk factor for acquiring COVID-19.

## Graphical abstract

**Figure:**
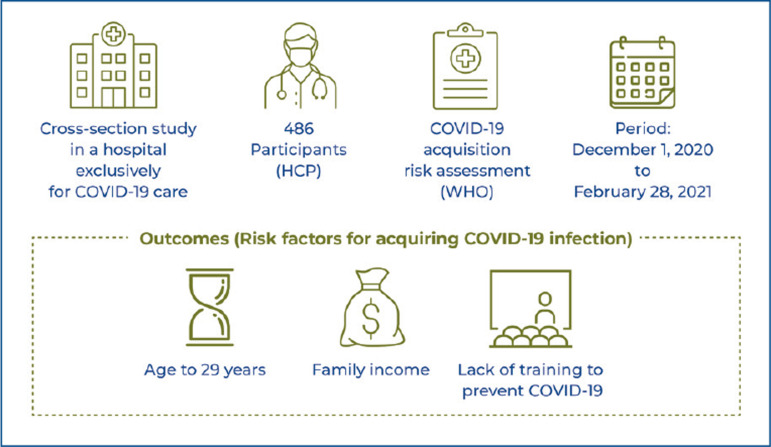

## INTRODUCTION

COVID-19 infection, caused by the SARS-CoV-2 virus, was declared a pandemic by the World Health Organization (WHO) on March 11, 2020. It is responsible for the greatest global health crisis, impacting the daily lives of individuals, health systems, and economic and social spheres, regardless of country, continent, ethnicity, or socioeconomic group.^(1,2)^

COVID-19 infection affects healthcare personnel (HCP), with cases of occupational infection in health services worldwide. This highlights the importance of applying infection prevention and control (IPC) measures to reduce infection risk among HCP.^(2-4)^ Cases of COVID-19 infection among HCP were reported between 2020 and 2021 in several countries. Specifically, 100,570 cases were reported in the USA on July 2020;^(5)^ 15,000 cases occurred among HCP in Italy;^(6)^ and 69,342 cases were reported in Mexico.^(7)^ According to the Ministry of Health of Brazil, 650,456 cases of suspected COVID-19 were reported in 2021, of which 153,247 (23.6%) were confirmed via diagnostic testing. The highest proportion of cases occurred among the following HCP categories: nurse technicians (45,631; 29.8%), nurses (25,853; 16.9%), and physicians (16,574; 10.8%).^(8)^

Understanding the epidemiology of SARS-CoV-2 infection, as well as its corresponding risk factors, is essential for implementing improvement strategies to ensure the safety of HCP and patients.^(2,6,8,9)^ The guiding question of this research was as follows: What is the risk of occupational infection in HCP exposed to SARS-CoV-2 in a specific hospital during care for patients with COVID-19?

## OBJECTIVE

This study aimed to verify the rate of COVID-19 infection among healthcare personnel at high and low risk of COVID-19 infection and identify the underlying risk factors.

## METHODS

This was a cross-sectional study with a quantitative approach carried out in a public hospital with 180 beds, 30 of which were infirmary beds and 150 beds were in the nursing intensive care unit (ICU), between December 1, 2020, and February 28, 2021. This study was approved by the Research Ethics Committee of *Secretaria Municipal da Saúde de São Paulo* (CAAE: 38453120.5.3001.0086; # 4.402.373).

The hospital was organized to care for patients with COVID-19, including reception flows and signage, manuals, standards, and care routines. Care routines included individual and collective HCP protection measures and educational materials on IPC, which are available as posters at the point of care and via the Internet. The educational materials included respiratory etiquette, hand hygiene, and the use of personal protective equipment (PPE).

The institutional protocol for all areas of care (ICU, ward, and diagnostic department) included the use of individual clothing for each HCP and the following PPE: PFF2/N95 type masks, goggles or face shields, isolation gowns, and new gloves for each procedure. All HCP working directly or indirectly in various departments should use all the recommended PPE when entering these areas.

The surgical mask was standardized to be universally used by all workers at the institution from entry to exit from the hospital, except in care areas where using a PFF2/N95 mask was mandatory. Initial training was made available for HCP on admission and later care for patients with COVID-19, as well as the use of PPE.

Healthcare personnel were invited to participate in the study. After clarifying the research objectives and methodology, all participants signed the free and informed consent form. In a specific room, they also completed the electronic self-administered form called “Assessment of the risk of exposure of HCP to the COVID-19 infection virus in health services” through the REDCap^®^ platform. Biosecurity measures were applied throughout the data collection process (hand hygiene and disinfection of materials and equipment used by the participants).

The instrument used was developed by the WHO to determine the risk classification of each HCP after exposure to a patient with COVID-19. It was translated into Brazilian Portuguese by the Pan-American Health Organization (PAHO/Brazil).^(10,11)^The instrument consisted of two parts. Part I contains the sociodemographic characteristics of the research participants, data on HCP training, information on the working time and training on COVID-19 infection, and the use of PPE. Regarding sociodemographic characteristics, salary was accounted for in the analysis; the minimum wage in the Brazilian economic system was U$ 233.98. Thus, the mensal payments of HCP were categorized by the number of times their salary was a multiple of the minimum wage (*e.g.*, 2 minimum wages means U$ 467.96/month). Part II is a questionnaire with questions related to the risk of HCP exposure to SARS-CoV-2 in the care of patients with COVID-19 infection at the institution (survey site). It contains seven components that evaluate independent dimensions: a) staying in the same household or traveling with or near (less than 1 meter) a confirmed case of COVID-19 infection, b) HCP category and place of activity, c) interaction with COVID-19-infected patient and date of first exposure to a confirmed COVID-19 infection case; d) activities performed with or presence during procedures with aerosol generation (PGA), direct contact with environmental surfaces or assistance care for a patient with COVID-19 infection in another health service; e) adherence to IPC measures: correct use of PPE, hand hygiene (HH) in the 5 moments (before touching a patient, before a procedure, after a procedure or body fluid exposure risk, after touching a patient, after touching a patient’s surroundings) and environmental hygiene (cleaning/disinfection) at least three times a day; f) regarding the correct use of PPE, the participant should answer “always as recommended” if used more than 95% of the time; “most of the time” means 50% or more, but not 100% of the time; “sometimes” means 20% to less than 50%, and “rarely” means less than 20%; saw accident with biological material in the care of a patient with COVID-19 infection (yes or no, type of accident); diagnosis of COVID-19 infection after starting work at the institution: yes or no (date, signs and symptoms, and type of test performed); and g) if the HCP has been vaccinated against COVID-19.

COVID-19 infections in HCP were confirmed with a positive polymerase chain reaction (PCR) test, regardless of whether they had signs or symptoms or had a positive serology result (IgM or IgG) with two or more symptoms characteristic of a COVID-19 infection and did not receive any doses of the vaccine. HCP were classified as not having COVID-19 if the PCR test was negative or if it was not performed, or if the serology result was positive but the HCP did not show signs or symptoms of COVID-19.

Regarding the risk classification of the HCP for SARS-CoV-2 infection, they were considered “high-risk” if they provided a negative answer to “Always, as recommended” to the questions in item 5 (IPC measures) or a “Yes” answer to the questions in item 6 (accident with biological material), and “low-risk” when their answer was affirmative to “Always, as recommended” to the questions in item 5 (IPC measures) and answered “No” to the questions in item 6 (accident with biological material).

Information on socioeconomic status, HCP training, working conditions, activities related to PGA, and increased risk of infection with SARS-CoV-2 were considered independent variables. The dependent variable was whether the HCP had a COVID-19 infection (yes or no) and, if so, what the level of risk of infection exists, whether high or low.

The proportion of HCP classified as being at high risk for COVID-19 infection was calculated, as well as the corresponding 95% confidence interval (95%CI). Socioeconomic data, HCP training, working conditions, and participation in PGA were described by occupational risk level group (low or high) and adjusted using binary logistic regression models with simple and multiple approaches to investigate their associations with infection risk. The model results are presented as estimated odds ratios (ORs), 95%CIs, and p-values.^(12,13)^

Data were collected and stored on the REDCap platform, and analyses were performed using the SPSS program with a significance level of 5%.

## RESULTS

A total of 737 HCP providing direct care to patients with COVID-19 participated in the study. However, 251 records were excluded for those HCP who did not answer the questions in item 5 about IPC measures.

For the final analysis, data from 486 HCP were considered, whose working time at the institution on the date of participation in the research was between zero and 283 days, with a median of 138.5 days (first quartile, 68 days; third quartile, 195 days). Most were women (357; 73.5%), with a mean age of 37.2 ± 8.2 years. The proportion of HCP who declared themselves as white (220; 45.3%) or brown (206; 42.4%) were similar. Also similar percentages between the extremes of the family income classification, with 97 HCP (20.0%) earning up to three minimum wages and 100 (20.6%) receiving nine minimum wages or more. In table 1, 496 health professionals are described instead of 486 because 10 health professionals worked in both the nursery and ICU.

**Table 1:** Characterization of health professionals participating in the study who worked in direct care for patients with COVID-19

Professional categories	n (%)
Physician	71 (14.6)
Nurse	75 (15.4)
Nursing technician	251 (51.6)
Physiotherapist	75 (15.4)
Speech therapist	9 (1.9)
Nutritionist	5 (1.0)
Years of experience	
<1	60 (12.3)
1-5	150 (30.9)
6-10	131 (27.0)
11-15	77 (15.8)
16-20	41 (8.4)
≥ 21	27 (5.6)
Workplace (n=496)*	
Nursery	138 (27.8)
Intensive care unit	357 (72.0)
Nutrition sector	(0.2)

* Operation in both units (n=10).

Most participants were from the HCP category of nurse technicians (251; 51.6%), followed by nurses and physiotherapists (75 each; 15.4% each), doctors (71; 14.6%), and other HCP such as speech therapists (9; 1.9%) and nutritionists (5; 1.0%). These HCP worked mainly in ICUs (357, 73.5%). Regarding the length of HCP experience, 281 (57.9%) had 1-10 years of experience in their healthcare area (Table 1).

Most HCP took a training course in caring for patients with COVID-19 (330; 67.9%). Moreover, 354 (70%) stated that they had received training for both putting on and taking off PPE.

Regarding adherence to IPC measures, research participants reported using the following PPE “always as recommended”: disposable gloves: 454 (93.4%), N95/PFF2 (or equivalent respirator): 459 (96.5%), face shield or goggles: 281 (57.8%), disposable apron: 460 (94.7%), and removing and changing their PPE: 377 (77.6%). Of the five HH moments, 434 (89.3%) mentioned performing them before and after touching a patient, 426 (87.7%) did them before performing any cleaning or aseptic procedure, and 348 (71.6%) performed them after touching surfaces around a patient with COVID-19. Regarding the cleaning and disinfection of high-contact surfaces, 304 (62.6%) participants stated that they were frequently decontaminated (at least three times a day).

Among the 59 HCP who had COVID-19, 57 were symptomatic. They experienced symptoms after starting work at the institution (median of 82 days and [Q1:39; Q3:131]), including the following: headache (46; 86.7%), muscle pain (34; 59.6%), fever (32; 56.1%), cough (30; 52.6%), loss of taste (29; 50.9%), loss of smell (28, 49.1%), fatigue (26; 45.6%), sore throat (20; 35.1%), shortness of breath (18; 31.6%), diarrhea (16; 28%), abdominal pain (8; 14%), nausea (8; 14%), and other symptoms (4; 7%).

Among the research participants, 57 (11.7%) underwent PCR for the diagnosis of COVID-19, of which 55 (96.5%) had a positive result. In addition, 15 (3.1 %) underwent serology, of which 13 (86.7%) were IgM-positive and 14 (93.3%) had positive IgG serology.

The final classification of COVID-19 infection among survey participants was as follows: 426 (87.7%) had no diagnosis of COVID-19 infection (positive serology without symptoms or PCR-negative/not performed), 59 (12.1%) with a confirmed diagnosis of COVID-19 infection (PCR-positive, regardless of whether they had symptoms, having positive serology with two or more characteristic symptoms, and did not receive any vaccine), and one (0.2%) with a suspected diagnosis (had characteristic symptoms and negative PCR performed outside the recommended period and did not perform serology).

During the course of this study, 140 (28.8%) HCP received a COVID-19 vaccine and participated in clinical research. The SINOVAC/Instituto Butantã vaccine was given to 136 (28.0%) HCP and the University of Oxford/AstraZeneca to four (0.8%); 116 (23.9%) received one dose and 24 (4.9%) received two doses.

Regarding the classification of the occupational risk level for COVID-19 infection, 207 HCP (42.6%) were considered low-risk, whereas 279 (57.4%) were identified as high-risk (Table 2). Of the HCP classified as high-risk, 32 (11.5%) had COVID-19; among those at low risk, 27 (13%) had COVID-19.

**Table 2:** Characterization of health professionals diagnosed with COVID-19 and occupational risk classification for SARS-CoV-2 infection

COVID-19 diagnosis	n (%)
No	426 (87.7)
Yes	59 (12.1)
Unconfirmed diagnosis of COVID-19	1 (0.2)
Occupational risk classification	
Low-risk	207 (42.6)
High-risk	279 (57.4)

Table 3 presents the analysis of the association between socioeconomic variables and occupational risk of exposure to SARS-CoV-2 among participants working in the direct care of patients with COVID-19 (n=486). Dividing into age groups of up to 29 years and 30 years or more, the chance of a high level of risk for HCP aged up to 29 years was estimated to be 2.5 times higher than the chance among HCPs aged 30 years or more (OR=2.51; 95%CI= 1.54-4.10; p<0.001).

**Table 3:** Association between sociodemographic characteristics of healthcare professionals assisting patients with COVID-19 and occupational risk in exposure to the SARS-CoV-2 virus

	Occupational risk classification		OR (95%CI)	p value
	Low-risk (n=207)	High-risk (n=279)		
Average (SD)	38.5 (7.9)	36.3 (8.2)	0.966 (0.945-0.988)	0.003
Age group (years), n (%)														
20-29 years (n=100)	26 (26.0)	74 (74.0)	2.51 (1.54-4.10)	<0.001
≥30 years (n=386)	181 (46.9)	205 (53.1)	1.00	---
Gender, n (%)				
Female (n=357)	158 (44.3)	199 (55.7)	1.00	---
Male (n=129)	49 (38.0)	80 (62.0)	1.30 (0.86-1.96)	0.217
Ethnicity, n (%)				
White (n=220)	97 (44.1)	123 (55.9)	0.42 (0.04-4.13)	0.459
Black (n=56)	22 (39.3)	34 (60.7)	0.52 (0.05-5.27)	0.576
Brown (n=206)	87 (42.2)	119 (57.8)	0.46 (0.05-4.46)	0.500
Yellow (n=4)	1 (25.0)	3 (75.0)	1.00	---
Monthly family income (basic salary), n (%)				
≤8 (n=386)	182 (47.2)	204 (52.8)	1.00	---
≥9 (n=100)	25 (25.0)	75 (75.0)	2.68 (1.63-4.39)	<0.001
Number of people living in the same house, n (%)				
≤3 (n=291)	123 (42.3)	168 (57.7)	1.00	---
≥4 (n=195)	84 (43.1)	111 (56.9)	0.97 (0.67-1.40)	0.860
Number of rooms in the house, n (%)				
≤5 (n=291)	126 (43.3)	165 (56.7)	1.00	---
≥6 (n=195)	81 (41.5)	114 (58.5)	1.07 (0.74-1.55)	0.700
Use public transport, n (%)				
No (n=253)	103 (40.7)	150 (59.3)	1.00	---
Yes (n=233)	104 (44.6)	129 (55.4)	0.85 (0.59-1.22)	0.382

SD: standard deviation; OR: odds ratio; 95%CI: 95% confidence Interval; Basic salary = U$ 233.98 (a basic salary ≤2 means ≤U$ 467.96).

There is evidence that the chance of a high level of occupational risk is lower in HCP with a family income of up to eight times the minimum wage compared with HCPs with a family income of nine times the minimum wage or more. That is, the chance of a high level of risk for HCPs with a family income of nine minimum wages was estimated to be 2.7 times higher the chance among HCPs with up to eight minimum salaries (OR=2.68; 95%CI= 1.63-4.39; p<0.001).

There was no association between risk level and sex (p=0.217), ethnicity (global p=0.815), number of people living in the same house as the HCP (p=0.860), number of rooms in the house (p=0.700), or use of public transportation (p=0.382). Table 4 presents the associations between the time of experience, work activities, and presence during procedures that generate aerosols according to the risk classification of exposure to SARS-CoV-2.

**Table 4:** Association between time of experience in the health area, work activities, and presence in procedures that generate aerosols and risk of exposure to the SARS-CoV-2 virus among health professionals in the care of patients with COVID-19

	Occupational risk classification		OR (95%CI)	p value
	Low-risk (n=207) n (%)	High-risk (n=279) n (%)		
Time of experience in the health área (years)				
<1 (n=60)	24 (40.0)	36 (60.0)	1.00	---
1-5 (n=150)	54 (36.0)	96 (64.0)	1.19 [0.64-2.19]	0.588
6-10 (n=131)	64 (48.9)	67 (51.1)	0.70 [0.38-1.30]	0.255
11-15 (n=77)	36 (46.8)	41 (53.2)	0.76 [0.38-1.50]	0.430
16-20 (n=41)	20 (48.8)	21 (51.2)	0.70 [0.31-1.56]	0.383
21 (n=27)	9 (33.3)	18 (66.7)	1.33 [0.51-3.46]	0.554
Received training prior to caring for patients with COVID-19				
No (n=156)	42 (26.9)	114 (73.1)	2.71 [1.79-4.11]	<0.001
Yes (n=330)	165 (50.0)	165 (50.0)	1.00	---
Received training on personal protective equipment				
No (n=103)	28 (27.2)	75 (72.8)	2.35 [1.46-3.79]	<0.001
Yes (n=383)	179 (46.7)	204 (53.3)	1.00	---
Health professional				
Physician (n=71)	14 (19.7)	57 (80.3)	1.00	---
Nurse (n=75)	42 (56.0)	33 (44.0)	0.19 [0.09-0.41]	<0.001
Nursing technician (n=251)	110 (43.8)	141 (56.2)	0.31 [0.17-0.59]	<0.001
Physiotherapist (n=84)	39 (46.4)	45 (53.6)	0.28 [0.14-0.59]	0.001
Nutricionist (n=5)	2 (40.0)	3 (60.0)	0.37 [0.06; 2.42]	0.298
Works in intensive care				
No (n=129)	59 (45.7)	70 (54.3)	1.00	---
Yes (n=357)	148 (41.5)	209 (58.5)	1.19 [0.79-1.79]	0.400
Participate in procedures that generate aerosols				
Orotracheal intubation				
No (n=123)	60 (48.8)	63 (51.2)	1.00	---
Yes (n=363)	147 (40.5)	216 (59.5)	1.40 [0.93- 2.11]	0.109
Nebulization				
No (n=367)	157 (42.8)	210 (57.2)	1.00	---
Yes (n=119)	50 (42.0)	69 (58.0)	1,03 [0.68- 1.57]	0.884
Open system airway suctioning				
No (n=300)	118 (39.3)	182 (60.7)	1.00	---
Yes (n=186)	89 (47.8)	97 (52.2)	0.71 [0.49- 1.02]	0.065
Sputum collection				
No (n=345)	148 (42.9)	197 (57.1)	1.00	---
Yes (n=141)	59 (41.8)	82 (58.2)	1.04 [0.70- 1.55]	0.831
Tracheostomy				
No (n=274)	110 (40.1)	164 (59.9)	1.00	---
Yes (n=212)	97 (45.8)	115 (54.2)	0.80 [0.55-1.14]	0.215
Bronchoscopy				
No (n=475)	201 (42.3)	274 (57.7)	1.00	
Yes (n=11)	6 (54.5)	5 (45.5)	0.61 [0.18-2.03]	0.422
Cardio-pulmonary resuscitation				
No (n=191)	94 (49.2)	97 (50.8)	1.00	---
Yes (n=295)	113 (38.3)	182 (61.7)	1.56 [1.08-2.26]	0.018
Other procedures				
No (n=474)	203 (42.8)	271 (57.2)	1.00	---
Yes (n=12)	4 (33.3)	8 (66.7)	1.50 [0.44-5.04]	0.514

OR: odds ratio; 95%CI: 95% confidence interval.

For HCP who did not participate in the initial training to work in the area of COVID-19-infected patients, the chance of a high level of risk of occupational COVID-19 infection was estimated to be 2.7 times higher the chance among HCP who participated in the training (OR=2.71; 95%CI= 1.79-4.11; p<0.001). The odds for a high level of occupation risk for COVID-19 for HCP who did not participate in training regarding the use of PPE was estimated at 2.3 times higher the chance among HCP who participated in the training (OR=2.35; 95%CI= 1.46-3.79; p<0.001).

Regarding HCP categories, the results indicate that the odds of a high level of risk are lower in nurses (OR=0.19; 95%CI= 0.09-0.41; p<0.001), nurse technicians (OR=0.31; 95%CI= 0.17-0.59; p<0.001), and physiotherapists (OR=0.28; 95%CI= 0.14-0.59; p=0.001) compared with medical HCP, and that does not differ for nutritionists (OR=0.37; 95%CI= 0.06-2.42; p=0.298). There was no evidence of an association between the level of risk and the work of HCP in the ICU (p=0.400).

It was also observed that only participating in the cardiopulmonary resuscitation procedure determined an increase in the risk among HCP, with an increase equivalent to 1.6 times higher odds (OR: 1.56; 95%CI= 1.08-2.26; p=0.018) (Table 4).

After adjusting for a simple approach, the associations between the level of occupational risk (low or high) and the characteristics of the participants were investigated using logistic models in a multiple approach with a stepwise method of selection of variables. After adjusting all variables with p<0.20 in the simple models, the final model was formed from the age and monthly family income of the HCP and the initial training to work in the care of patients with COVID-19 infection (Table 5). Thus, the chance of a high occupational risk for HCP aged up to 29 years was estimated to be 2.7 times higher than that among HCP aged 30 years or older (OR=2.70; 95%CI= 1.63-4.47; p<0.001). For HCP with a monthly family income above eight minimum wages, the risk was estimated to be 1.8 times the chance among HCP with a monthly family income of up to eight minimum wages (OR=1.84; 95%CI= 1.07-3.16; p=0.027). Finally, HCP who did not participate in the initial training to work in the care of patients with COVID-19 had a high occupational risk estimated at 2.4 times the chance of HCP who received the training.

**Table 5:** Multiple logistic regression model for associations of characteristics of health professionals in the care of patients with COVID-19 with level of occupational risk (low or high)

Variable	OR (95%CI)	p value
Age group		
20-29 years (n=100)	2.70 [1.63; 4.47]	<0.001
≥30 years (n=386)	1.00	---
Family Monthly Income (Minimum wage)		
≤8 Minimum wage (n=386)	1.00	---
>8 Minimun wage (n=100)	1.84 [1.07; 3.16]	0.027
Received training prior to caring for COVID-19 patients		
No (n=156)	2.39 [1.53; 3.75]	<0.001
Yes (n=330)	1.00	---

OR: odds ratio; 95%CI: 95% confidence interval; Minimum wage = U$ 233,98 (*e.g*,: ≤8 means ≤U$ 1.871.84).

## DISCUSSION

The results of this study conducted in our hospital indicated that 59 (12.1%) HCP who worked in direct patient care had a positive diagnosis of COVID-19. This study began before the national vaccination campaign for COVID-19 infection launched in Brazil on January 17, 2021. Therefore, it was not possible to assess the impact of the vaccine on the risk of acquiring SARS-CoV-2 infection.

Most HCP (57.4%) had a high occupational risk of SARS-CoV-2 infection. Healthcare personnel with high and low occupational risk for SARS-CoV-2 reported having had COVID-19, but it was not possible to identify the place of acquisition of the virus, such as in the work environment or within the community.

The following variables were considered to have a greater chance of imparting high occupational risk for SARS-CoV-2 among the survey participants: up to 29 years of age, monthly family income greater than eight minimum wages, and not participating in the initial training to act in the care of patients with COVID-19.

In a study involving healthcare workers, the results were extracted from a database provided by the Center for Strategic Information on Health Surveillance of Salvador, where 74.8% of frontline HCP tested positive for COVID-19 infection.^(14-16)^ The rate of HCP who tested positive for COVID-19 in our study was lower than in other studies. This difference was possibly due to the training and correct use of PPE by most of the HCP in our study. Although 66% of the HCP underwent a training course in the aforementioned study, only 31% reported adhering to the use of PPE. Among 847 HCP from a general hospital in Kuwait, 20.5% had COVID-19 from August to October 2020, a rate higher than in our survey. This difference may be associated with several factors, such as the community transmission rate and the adoption of preventive measures such as the isolation of these HCP (even distancing them from their family), the use of PPE, the use of protocols developed in hospitals to control the spread of the disease, risk of infection, and vaccination coverage.^(9)^Other studies showed a COVID-19 infection rate similar to that described in this study, such as a systematic review with a meta-analysis of 97 articles that reported COVID-19 infection rates among HCP was 11%, as well as a study conducted in a hospital in Oman that reported a rate of 10.6%.^(3,14)^

Variations in the rate of infection among HCP may be related to the diagnostic performance of the RT-PCR test, which may be influenced by factors such as the origin of the biological sample and the time after the onset of symptoms at the time of collection. In addition, it is necessary to consider that, in the RT-PCR test, it is possible to obtain an initial negative result in individuals with COVID-19 if samples are collected at an early or late stage of infection.^(15,16)^

Regarding the association between age and the risk of COVID-19 infection described in many studies, it has been reported that higher levels of risk are associated with older HCP, which is inconsistent with our results. In a study involving 422 HCP in Ethiopia, it was concluded that the probability of infection was 87 times lower among HCP aged 35-44 years than those aged 18-24 years. This evident variation in results regarding the possible impact of age on the risk of SARS-CoV-2 infection indicates the need for further studies with adequate designs to understand this association better.^(1,17)^

Our findings regarding the impact of family income on the increased risk of infection among HCP differ from those of other studies, which claim that people with lower incomes and in situations of social vulnerability are the most exposed to COVID-19 infection.^(18)^ However, in our study, it is possible to justify this divergence when considering the medical category with a high occupational risk for COVID-19 (univariate analysis), whose salaries are higher than those offered to other HCP.

The higher risk of infection among the physicians described here was not confirmed in the multivariate analysis. However, other studies conducted in Portugal confirmed this result, indicating a significantly higher risk among physicians caring for patients with COVID-19 than other HCP categories.^(19)^

The importance of using PPE and training regarding the prevention and reduction of risk among HCP who work in the direct care of patients with COVID-19 described in our results is similar to that presented by other researchers.^(19,20)^Among these, a rapid review reported an association between the reduction in the risk of HCP infection and the accomplishment of training and education.^(20)^ In another study conducted at a hospital in São Paulo, Brazil, the training of a multidisciplinary team contributed to reducing the risk of infection. These results highlight the importance of having well-established guidelines on the use of PPE, clear recommendations on the medical certificate policy for all HCP diagnosed with COVID-19, and strong supervision for compliance with the use of PPE. This need for supervision, in addition to the continuous monitoring and evaluation of the proper use of PPE, could also be a recommendation for hospitals where our study was conducted, as the results indicate the importance of training to reduce the occupational risk of COVID-19 infection.^(20,21)^ In addition, the findings described here are important for planning actions to better control and manage COVID-19 in the hospitals studied.

As a limitation of this research, we cite the study design, which was a prevalence study in a specific hospital to treat patients with COVID-19 in a certain epidemiological situation. Hence, our results cannot be generalized. In addition, the participants only comprised HCP who directly assisted patients with COVID-19. The use of a self-reported questionnaire could also be a limiting factor, as the answers given may not reflect the actual practice of HCP, and the answers given may be based on the knowledge of what would be most appropriate in pandemic situations, such as COVID-19. Other limitations include the low COVID-19 vaccine compliance since the COVID-19 vaccine in Brazil, which started only in January 2021. Moreover, the lack of evidence of an association between the use of public transport and a risk of acquiring COVID-19 infection is hampered by the non-possibility of verifying the use of a mask by the HCP. Finally, the effects of SARS-CoV-2 variants (such as Omicron, Delta, etc.) were not evaluated during the study period.

## CONCLUSION

The number of healthcare personnel who had COVID-19 was low, and both healthcare personnel classified as high and low risk had COVID-19 infection. The independent risk factors for healthcare personnel classified as being at high risk of COVID-19 infection were younger age (under 30 years of age) with a higher monthly income. Factors such as participating in training before joining the COVID-19 patient care team and infection prevention and control measures (proper use of personal protective equipment, hand hygiene, and environmental hygiene) were fundamental for healthcare personnel to be considered at low risk for SARS-CoV-2 infection. Therefore, encouraging training for occupational infection prevention is important to reduce the impact of infectious diseases on healthcare personnel, hand hygiene, especially younger health professionals.

## References

[1] Pijls BG, Jolani S, Atherley A, Derckx RT, Dijkstra JI, Franssen GH (2021). Demographic risk factors for COVID-19 infection, severity, ICU admission and death: a meta-analysis of 59 studies. BMJ Open.

[2] Mendes TT, Ribeiro AP, Andrade CA, Bastos PK, Pádue PD (2021). Investigação epidemiológica de COVID-19 infection relacionada ao trabalho em trabalhadores de saúde: experiência do Cerest Salvador. Rev Baiana Saúde Pública.

[3] Gómez-Ochoa SA, Franco OH, Rojas LZ, Raguindin PF, Roa-Díaz ZM, Wyssmann BM (2021). COVID-19 INFECTION in health-care workers: a living systematic review and meta-analysis of prevalence, risk factors, clinical characteristics, and outcomes. Am J Epidemiol.

[4] Kahlert CR, Persi R, Güsewell S, Egger T, Leal-Neto OB, Sumer J (2021). Non-occupational and occupational factors associated with specific SARS-CoV-2 antibodies among hospital workers - a multicentre cross-sectional study. Clin Microbiol Infect.

[5] Hughes MM, Groenewold MR, Lessem SE, Xu K, Ussery EN, Wiegand RE (2020). Update: characteristics of health care personnel with COVID-19 INFECTION. United States, February 12-July 16, 2020. MMWR Morb Mortal Wkly Rep.

[6] Garzaro G, Clari M, Ciocan C, Grillo E, Mansour I, Godono A (2020). COVID-19 infection and diffusion among the healthcare workforce in a large university-hospital in northwest Italy. Med Lav.

[7] Robles-Pérez E, González-Diaz B, Miranda-García M, Borja-Aburto V (2021). Infection and death by COVID-19 INFECTION in a cohort of healthcare personnel (HCP) in Mexico. Scand J Work Environ Health.

[8] Brasil, Ministério da Saúde, Secretária de Vigilância em Saúde (2021). Boletim Epidemiológico Especial - Doença pelo Coronavírus COVID-19. Semana Epidemiológica 48 (28/11/21 a 04/12/2021).

[9] Youha SA, Alowaish O, Ibrahim IK, Alghounaim M, Abu-Sheasha GA, Fakhra Z (2021). Factors associated with COVID-19 infection amongst healthcare personnel (HCP) in a COVID-19 INFECTION designated hospital. J Infect Public Health.

[10] World Health Organization (WHO) (2020). Risk assessment and management of exposure of health care workers in the context of COVID-19 infection Interim guidance 19 March 2020.

[11] Organização Pan-Americana da Saúde (OPAS/OMS Brasil) (2020). Avaliação de risco e gerenciamento da exposição de profissionais de saúde no contexto da COVID-19. Orientação provisória 19 de março de 2020.

[12] Hosmer DW, Lemeshow S (2004). Applied Logistic Regression.

[13] Altman DG (1991). Practical Statistics for Medical Research.

[14] Al-Siyabi N, Al-Lawati A, Al-Lawati M, Al-Wahibi I, Al-Nasseri M, Al-Maashari A (2021). Characteristics and immunoglobulin G seropositivity among COVID-19 INFECTION positive healthcare personnel (HCP) in a tertiary care hospital in Oman. Oman Med J.

[15] To KK, Tsang OT, Leung WS, Tam AR, Wu TC, Lung DC (2020). Temporal profiles of viral load in posterior oropharyngeal saliva samples and serum antibody responses during infection by SARS-CoV-2: an observational cohort study. Lancet Infect Dis.

[16] Arevalo-Rodriguez I, Buitrago-Garcia D, Simancas-Racines D, Zambrano-Achig P, Del Campo R, Ciapponi A (2020). False-negative results of initial RT-PCR assays for COVID-19: a systematic review. PLoS One.

[17] Antnafie SA, Anteneh DA, Yimenu DK, Kifle ZD (2021). Assessment of exposure risks to COVID-19 INFECTION among frontline health care workers in Amhara Region, Ethiopia: a cross-sectional survey. PLoS One.

[18] Estrela FM, Soares CF, Cruz MA, Silva AF, Santos JR, Moreira TM (2020). Covid-19 Pandemic: reflecting vulnerabilities in the light of gender, race and class. Cien Saude Colet.

[19] Sousa-Uva M, Sousa-Uva A, Serranheira F (2021). Prevalence of COVID-19 in health professionals and occupational psychosocial risks. Rev Bras Med Trab.

[20] Chou R, Dana T, Buckley DI, Selph S, Fu R, Totten AM (2020). Epidemiology of and risk factors for coronavirus infection in health care workers. Ann Intern Med.

[21] Buonafine CP, Paiatto BN, Leal FB, Matos SF, Morais CO, Guerra GG (2020). High prevalence of COVID-19 infection among symptomatic healthcare personnel (HCP) in a large university tertiary hospital in São Paulo, Brazil. BMC Infect Dis.

